# Supplementary material for: Exploring unintended pregnancy journeys among women with psychiatric vulnerability using interpretative phenomenological analysis
Source: PLoS One. 2026 Feb 23;21(2):e0329516. doi: 10.1371/journal.pone.0329516 (PMC12928457; doi:10.1371/journal.pone.0329516)
Supplement: S1 Appendix — (DOCX) [file pone.0329516.s001.docx]

**Appendix 1 – topic lists

Antepartum interview**

**Grandtour: could you tell me something about your pregnancy so far?**

1. Wish to have children

- Background wish to have children
- Role psychological complaints in getting pregnant unintended (mechanism)
  *- Could you tell us something about why you attend the POP-outpatient clinic?*
- Experience of an unintended pregnancy (meanwhile maybe intended?)

2. Theoretical framework pregnancy

- Fantasy pregnancy 🡪 fantasies about the baby
- Phantom pregnancy 🡪 where are you afraid of?
- Actual pregnancy 🡪 safety/security
  *- Experience movements of the baby*

3. Environment

- *Migration: born in which country/how long have you been living in NL/experience of cultural differences regarding mental health and/or unintended pregnancies?*
- Role environmental/cultural/religious background (in relation to stigma)
   *- Do you feel like your diagnosis comes with a label (stigma)?
  - Did your diagnosis influence your wish to have children?*
- Role environmental/cultural/religious background in relation to freedom of choice?
  *- What did you discuss with friends/family about your diagnosis and/or unintended pregnancy?*
- Role caregiver (tips)
- Wish for support during pregnancy
  *- Does the psychological counseling/guidance meet your wishes and needs?
  - Social network/experiences other mothers/fellow patients*
  *- Other people pregnant in own social network?*

4. Questions role psychiatric issues in upcoming motherhood:

- Intergenerational issues
- Complex grief
- Having parents with a psychiatric vulnerability
- Inheritance/genetics
- Having parents with psychiatric vulnerability or substance abuse
  *- Do you want to share something about the family that you grew up in?*
- Role medication and possible worries?
- Substance use (does it influence the way you think about the pregnancy)
- Role psychiatric complaints. *Fear or anxiety?*
- Repair own trauma
- Disorganised/problems executive functions

5. Transition to motherhood

- *Do you have an idea about what kind of mother you want to be?*
- *Are you looking forward to being a mother?*

**Postpartum conversation**

**Grand tour: Can you tell me what it is like to be a mother?**

1. Referring back: Has your intention regarding pregnancy / becoming a parent changed?
2. Theoretical framework: the three babies
   1. Fantasy baby
   2. Phantom baby
   3. Actual baby
3. Mother–child bonding
4. Birth experience
5. Desired support in the postpartum period (friends, family, social domain?)

**Partner**

**Grand tour: Can you tell me what it is like to be a parent?**

1. Referring back: Has your intention regarding pregnancy / becoming a parent changed?
2. Theoretical framework: the three babies
   1. Fantasy baby
   2. Phantom baby
   3. Actual baby
3. Parent–child bonding
4. Role of psychological problems in unintended pregnancy (mechanism)
5. Desired support in the postpartum period (friends, family, social domain?)
