# Supplementary material for: Exploring unintended pregnancy journeys among women with psychiatric vulnerability using interpretative phenomenological analysis
Source: PLoS One. 2026 Feb 23;21(2):e0329516. doi: 10.1371/journal.pone.0329516 (PMC12928457; doi:10.1371/journal.pone.0329516)
Supplement: Attachment — Submitted filename: Rebuttal letter.docx [file pone.0329516.s003.docx]

We thank the reviewers for their time and effort in providing constructive feedback on our manuscript. We have taken the opportunity to clarify several issues and feel the manuscript has benefitted from this, from our point of view.
We provide point-by-point responses to each of their comments below. When we added or adjusted text based on the feedback in the manuscript, we have indicated this in the responses below by putting the text in italic. **EDITOR COMMENTS**

**Journal requirements:**

- Please ensure that your manuscript meets PLOS ONE's style requirements, including those for file naming. The PLOS ONE style templates can be found at
  <https://journals.plos.org/plosone/s/file?id=wjVg/PLOSOne_formatting_sample_main_body.pdf> and
  [https://journals.plos.org/plosone/s/file?id=ba62/PLOSOne_formatting_sample_title_authors_affiliations.pdf](https://track.editorialmanager.com/CL0/https:%2F%2Fjournals.plos.org%2Fplosone%2Fs%2Ffile%3Fid=ba62%2FPLOSOne_formatting_sample_title_authors_affiliations.pdf/1/010f019a94f2413d-ef203f2b-6ec2-4aa1-a2ea-b6f73f46a25b-000000/tejgGA_qvjAVlYaKZCBZJwSHdyqA2kymHvrhC47QX0E=237)
  - Thank you for your feedback and apologies for the oversight. We have adjusted the format following the guidelines provided.
- Thank you for stating the following financial disclosure:
  [This research was funded by ZonMw, grant number 554002007]. 
  Please state what role the funders took in the study.  If the funders had no role, please state: "The funders had no role in study design, data collection and analysis, decision to publish, or preparation of the manuscript."
  If this statement is not correct you must amend it as needed.
  Please include this amended Role of Funder statement in your cover letter; we will change the online submission form on your behalf.
  - Thanks for this remark. The aforementioned statement was added to the manuscript at page 39 line 995-996
    *"The funders had no role in study design, data collection and analysis, decision to publish, or preparation of the manuscript."*
- In the online submission form, you indicated that [The data that support the findings of this study are available from the corresponding author upon reasonable request.].
  All PLOS journals now require all data underlying the findings described in their manuscript to be freely available to other researchers, either 1. In a public repository, 2. Within the manuscript itself, or 3. Uploaded as supplementary information.
  This policy applies to all data except where public deposition would breach compliance with the protocol approved by your research ethics board. If your data cannot be made publicly available for ethical or legal reasons (e.g., public availability would compromise patient privacy), please explain your reasons on resubmission and your exemption request will be escalated for approval.
  - The dataset for this study consists of qualitative interview transcripts containing rich, detailed narratives. Even with removal of direct identifiers, the contextual details pose a substantial risk of deductive disclosure and potential re-identification of participants. Because of these ethical constraints, publicly sharing the full transcripts would breach the confidentiality requirements approved by our institutional research ethics board. However, the metadata will be made available.
    Page 39 line 981-991 *The metadata for this study have been made publicly available to facilitate transparency and initial exploration (https://doi.org/10.34894/LISNEO). Access to the full database, which contains detailed information and study results, is restricted and will breach the confidentiality requirements approved by our institutional research ethics board, and requires explicit permission, due to the qualitative nature of the data. The database contains confidential, personal, and sensitive information that could compromise participant privacy, particularly given the inclusion of individuals from vulnerable communities. In accordance with the consent form, only disguised extracts may be used in scientific publications. Researchers interested in accessing the full database must contact the local review committee at OLVG (ACWO) via email (acwo@olvg.nl), which, in accordance with current laws and regulations, will review requests to ensure data privacy and ethical standards are maintained. The ACWO operates under the supervision of the Board of Directors of OLVG.*
- Your ethics statement should only appear in the Methods section of your manuscript. If your ethics statement is written in any section besides the Methods, please move it to the Methods section and delete it from any other section. Please ensure that your ethics statement is included in your manuscript, as the ethics statement entered into the online submission form will not be published alongside your manuscript.
  - The ethics have been moved to page 9 line 227 to 231 within the methods section.
    *This study was performed in accordance with the principles of the Declaration of Helsinki. The Medical Ethics Research Committee of the United Nieuwegein declared the study as not part of the Human Subjects Research Law, protocol code W21.304 and date of declaration 07-01-2022. Local approval was granted by the accredited local review board on 28-04-2022.*
- If the reviewer comments include a recommendation to cite specific previously published works, please review and evaluate these publications to determine whether they are relevant and should be cited. There is no requirement to cite these works unless the editor has indicated otherwise.
  - Thank you for your suggestion.

**Reviewers' comments:**

**Comments to the Author**

1. Is the manuscript technically sound, and do the data support the conclusions?

The manuscript must describe a technically sound piece of scientific research with data that supports the conclusions. Experiments must have been conducted rigorously, with appropriate controls, replication, and sample sizes. The conclusions must be drawn appropriately based on the data presented.

Reviewer #1: Partly

Reviewer #2: Partly

2. Has the statistical analysis been performed appropriately and rigorously?

Reviewer #1: N/A

Reviewer #2: N/A

3. Have the authors made all data underlying the findings in their manuscript fully available?

The [PLOS Data policy](http://track.editorialmanager.com/CL0/http:%2F%2Fwww.plosone.org%2Fstatic%2Fpolicies.action%23sharing/1/010f019a94f2413d-ef203f2b-6ec2-4aa1-a2ea-b6f73f46a25b-000000/Qnh_7TH8-tMLmhbI2fVWxKDcepoZrldj6MAQ-5FtTDE=237) requires authors to make all data underlying the findings described in their manuscript fully available without restriction, with rare exception (please refer to the Data Availability Statement in the manuscript PDF file). The data should be provided as part of the manuscript or its supporting information, or deposited to a public repository. For example, in addition to summary statistics, the data points behind means, medians and variance measures should be available. If there are restrictions on publicly sharing data—e.g. participant privacy or use of data from a third party—those must be specified.

Reviewer #1: Yes

Reviewer #2: No

4. Is the manuscript presented in an intelligible fashion and written in standard English?

PLOS ONE does not copyedit accepted manuscripts, so the language in submitted articles must be clear, correct, and unambiguous. Any typographical or grammatical errors should be corrected at revision, so please note any specific errors here.

Reviewer #1: Yes

Reviewer #2: Yes

5. Review Comments to the Author

Please use the space provided to explain your answers to the questions above. You may also include additional comments for the author, including concerns about dual publication, research ethics, or publication ethics. (Please upload your review as an attachment if it exceeds 20,000 characters)

**Reviewer #1: 
Abstract**

- In the results section, rather than describing the themes, I recommend summarizing the key findings or main takeaways to provide a clear overview.
  - Thank you for your suggestion. We have adapted the abstract as recommended.
    Page 2 line 36-45:
    *Women with psychiatric vulnerabilities described unintended pregnancies as complex events, often marked by ambivalent pregnancy intentions, concerns about generational trauma, and fears about parental adequacy. The pregnancies triggered heightened psychiatric symptoms, resurfacing childhood memories, and concerns about stigma, yet also motivated participants to seek support from mental health professionals and trusted others. Women adopted coping strategies such as focusing on the future, seeking distraction, and accepting support to manage emotional challenges. Across pregnancy and postpartum, many participants reported developing strong prenatal and postnatal bonding with the newborn, creating safety nets, and making intentional behavioral changes to support their babies. For several women, the unintended pregnancy ultimately fostered personal growth and contributed to an improvement in mental well-being.*

**Background**

- Line 93-97 should be rephrased to improve clarity
  - Thank you for the suggestion. We hope the following adaptations offer sufficient clarification.
    Page 4-5 line 103-109:
    *However, these findings are based on quantitative studies, which do not explore the individual experiences among pregnant women and their partners. In contrast, some qualitative studies have explored the experiences of UPs of women without psychiatric vulnerability. These studies describe pregnancy intentions as complex processes influenced by an interplay of both extrinsic factors, such as social, financial and relationship status, as well as intrinsic factors, such as the emotional and mental health status of the pregnant woman [21, 22].*
- The background section would benefit from additional depth, including evidence from previous studies and a more detailed explanation of the challenges faced by women with UPs.
  - Thank you for your suggestion, we now added more depth and evidence to the challenges faced by women with psychiatric vulnerabilities and UPs.
    Page 4 line 84-90
    *Unintended pregnancies (UPs, i.e. pregnancies that are mistimed and/or unwanted) account for up to 50% of all pregnancies worldwide [1]. UPs have a tremendous impact on pregnant women, their newborns and society, as they are associated with a higher risk for perinatal depression and lower birth weight, impaired parent‒child interactions, increased parenting stress, and a substantial social and financial burden [2-7].*
    Page 4 line 93-102
    *Patients with psychiatric vulnerability can experience difficulties in adhering to contraceptives, planning and reproductive autonomy [15-19]. For example, women with schizophrenia or bipolar disorders may suffer from cognitive and behavioral alterations that affect their reproductive planning and decisions. Studies found an association between depressive symptoms, or depressive symptoms co-occurring with stress, and inconsistent contraceptive use [15-19]. Stress and depressive symptoms may influence processes such as decision-making, risk assessment or increased side effects of contraceptives and therefore cause discontinuation [19]. Finally, one study found that women pertaining to a marginalized group and with a low socioeconomic status were more at risk for developing depression symptoms as well as UPs than women of majority or privileged groups. [20].*
- The study objectives (line 103-109) should be stated more clearly
  - Page 5 line 116-120
    We have adapted and clarified the aims.
    *This study aims to explore how women with psychiatric vulnerability experience unintended pregnancies. It seeks to examine the circumstances surrounding the UP, including how it occurred and which factors influenced the decision to continue the pregnancy. Other objectives are the experience of the mother-child bonding during pregnancy and the transition to parenthood. When possible, partners of the participants were also interviewed.*

**Methods**

- The manuscript appear to follow the JARS-Qual checklist instead of the COREQ or SRQR checklist, which are required by PLOS ONE. Please ensure compliance with journal guidelines.
  - Thank you for your comment. We have reviewed the checklists and we adhered to COREQ beside for one point: feedback from participants on the findings. We have discussed this in the limitations. The COREQ checklist is added as attachment.
    Page 6, line 143-144
    *The consolidated criteria for reporting qualitative research (COREQ) checklist was followed to assure transparency.*

Page 9 line 247-248
*The average duration of the antepartum interviews was 45 minutes and of the postpartum interviews was 48.6 minutes.*
Page 32, line 818-819
*While participants were offered to review the transcription and summary of their interview, they were not involved in the analysis and writing of the results*.

- Clarify what information was included in the patient files (line146) and how this data was analyzed.
  - Thank you for this remark. No information from the patients’ files was extracted or analyzed. All data were derived from the interviews. We understand that the term ‘patient file’ in line 156 can lead to misunderstanding. It has now been adapted.
    Page 7, line 179
    *When pregnant women agreed to participate, patient information documents and informed consent files were sent via email.*
- Consider providing the interview guide as a supplementary file
  - Thank you for your attentive remark. The interview guide was added as an appendix (see page 47-48).
- Explain the process for translating the interviews conducted in Dutch into English
  - Thank you for this important remark. We elucidated the process of translation.
    Page 8 line 213-215
    *As the researchers speak both Dutch and English, no translation was necessary and the analysis was performed in the same language as the interview was performed. The selected quotes for the manuscript were translated by the researchers.*
- Provide details on how demographic data were analyzed.
  - Thank you for requesting this clarification. We have not performed statistical analysis as it was not one of the aims of our research, we have only reported an overview.
    Page 8 line 206-207
    *Demographic data were described per individual patient and analyzed descriptively, using mean or number for group summaries.*

**Results**

- In the demographics section:
  - Include the attrition rate for the second round of interviews
    - Thanks for your suggestion, we calculated and added the attrition rate.
      Page 9 line 228-229
      *The attrition rate was 45.45%.*
  - Add mean age and key participant characteristics in the text.
    - Thank you for your suggestion.
      Page 9 line 237-239
      *The mean age of the participants was 32.3 years, of the partners was 29.5 years, most of them were primiparous except for one. Five women were born in the Netherlands, four in other countries (North America, Europe and South America) and the two partners in the Netherlands.*
- Line 193-198 – recommend removing this section
  - Page 12 line 254-259
    We removed the section to avoid redundancy.
- Line 262-263 – Recommend removing this section
  - Page 12 line 265-267
    We removed the section to avoid redundancy.
- Theme 1. While the results provide useful context, consider making this section more concise.
  - Thank you for the feedback, we condensed theme 1.
    - Page 13 Line 269-274 have been condensed
      *Most participants actively tried to avoid pregnancy by using contraception, such as copper intrauterine device, a birth control app or condoms. In these instances the discovery of the pregnancy was marked by astonishment.*
    - Page 13, Line 278-283
      *Other participants had misconceptions about their own fertility, due to older age or eating disorder and therefore did not use contraceptives. Participant 2 further commented that her pregnancy also caused relief: now that she had conceived, she was positively fertile.*
    - Page 12 Line 287:
      *Beside participant 2, no other participant indicated that her psychiatric vulnerability was related to the UP.*
    - Page 13 Line 290-294
      *The pregnancy intentions of several participants were ambivalent. This was illustrated by contradictory behavior toward preventing a pregnancy. For example participant 2 used folic acid but at the same time did not wish to become pregnant. The fear of consciously considering motherhood contributed to this ambivalence; participants 2, 3 and 7 were happy that the UP itself had prevented them from making the decision.*
    - Page 13 Line 313-317
      *It may be interpreted that making the decision to become a mother was something participant 3 did not want to do herself. Participant 7 echoed this experience by commenting that she did not want to make the decision to become a mother unless she was sure she would be a good mother. The UP had cleared the road for her on that account, as now she did not have to take on the responsibility of deciding. More participants described the pregnancy as something happening to them, instead of an act in which they were involved.*
    - Page 16 Line 359-360
      *Another participant used various words, which gave a negative tone to the discovery.*
    - Page 17 Line 380-381
      *Seven participants considered being in a stable relationship to be an important condition for pregnancy; the possibility of becoming a single mother was negatively seen.*

**Discussion**

- The “Main Findings” section should be condensed and integrated with “Interpretation of findings”.
  - Thank you we have condensed and integrated these sections. As it is quite a lengthy section despite the adaptations, we have not added it here. You can find the adapted section on pages 29-30, line 726-796.
- I suggest removing the subheading and presenting the content under a single “Discussion” heading.
  - We removed the subheadings.
- Include practical recommendations to strengthen the discussion.
  - Thank you for your suggestion. We have clarified some practical recommendations in the discussion.
    Page 37 line 933-947
    *The current clinical interventions available for this subgroup of women with psychiatric vulnerability in relation to UPs are limited. A scoping review on the transition to motherhood previously issued a warning that it would be a waste not to utilize the potential for growth during the transition to motherhood [60]. Based on our findings, HCPs should routinely screen for pregnancy intention in women with psychiatric vulnerabilities and offer counseling to explore possible ambivalences and consider the social conditions and possible previous traumatic experiences. Furthermore, they should encourage the involvement of partners and other pillars of support, and discuss possible emotions regarding bodily sensations of the baby or visualization thereof via the ultrasound. Initially unplanned but finally welcomed pregnancies could provide a window of opportunity to obtain appropriate psychiatric healthcare and tackle transgenerational patterns.*
- Move the “Strengths & Limitations” section immediately before the conclusion and make it more concise. Combine the “Implications for Future Research” with “Strengths & Limitations”, and shorten this combined section.
  - Thank you for your comment. We moved the Strengths and Limitations, combined with implications for future research and shortened both sections. The adaptations can be found on page 32 to page 36.

**Reviewer #2:**

**Introduction:**

- The introduction introduces the reader to the state of the literature about unintended pregnancies and provides a global statistic which highlights the significance of the topic. In line 88-90, the adverse effects of psychiatric vulnerability were described, however, the authors do not describe the mechanism by which these psychiatric vulnerabilities contribute to increased risk of UPs and among which populations (if there are existing disparities).
  - Page 4 line 93-102
    Thank you for your suggestion, we now elucidated these mechanisms according to the available current literature. We specified in which type of patients the current literature describes any disparity.
    *Patients with psychiatric vulnerability can experience difficulties in adhering to contraceptives, planning and reproductive autonomy [15-19]. For example, women with schizophrenia or bipolar disorders may suffer from cognitive and behavioral alterations that affect their reproductive planning and decisions. Studies found an association between depressive symptoms, or depressive symptoms co-occurring with stress, and inconsistent contraceptive use [15-19]. Stress and depressive symptoms may influence processes such as decision-making, risk assessment or increased side effects of contraceptives and therefore cause discontinuation [19]. Finally, a study found that women pertaining to a marginalized group and with a low socioeconomic status were more at risk for developing depression symptoms as well as UPs than women of majority groups or privileged groups [20].*
- In line 94, review language used when attributing UP to women experiencing psychiatric vulnerability. For example, "...UP among women..." would be more appropriate as the term emphasizes the collective.
  - Page 4 line 104
    Thank you for the suggestion we have changed the term with “*UP among women*” as advised.
- In line 103-104, there appears to be a misalignment between the gap stated in line 102; ongoing pregnancies is a new term that was not discussed elsewhere in the introduction nor was it defined. To strengthen this section, describe the state of the literature on ongoing pregnancies once this term has been defined as you have done with UP. The gap you have identified should reflect that to date, there has not been a study that explores how women with psychiatric vulnerability experience UP and ongoing pregnancy.
  - Page 5 line 121
    The term unintended ongoing pregnancies was redundant. By ongoing we meant unintended pregnancies which have not been interrupted. This is the same definition as unintended pregnancies. Thank you for noticing, we have now removed the term ‘ongoing’ to avoid confusion and redundancy.
- In line 104, the objectives are unclear. The term "besides" is informal. Please review the manuscript for similar instances. It appears that your objectives are to (1) identify and describe how UP occurs among women with psychiatric vulnerability and (2) factors that influence decisions around maintaining the pregnancy.
  - Thank you for the suggestion, we have adapted and clarified the aims.
    Page 5 line 116-120
    *This study aims to explore how women with psychiatric vulnerability experience unintended pregnancies. The objectives are to (1) identify and describe how UP occurs among women with psychiatric vulnerability and (2) factors that influence decisions around maintaining the6 pregnancy.* *Other objectives concern the mother-child bonding during pregnancy and the transition to parenthood. When possible, partners of the participants will also be interviewed.*
- In line 107-109, you have introduced a new gap in the literature. The first time it is mentioned should be earlier in the background, not as part of the aims. In doing so, justification for the objectives is substantiated.
  - Thank you for the suggestion, we have mentioned this important gap in the research earlier in the text.
    Page 4-5 line 112-114
    *Partners’ perspectives are often overlooked in research on UPs, even though they may play an important role in the decision-making process [31].*
- All persons may not be familiar with family planning which was mentioned twice in the introduction. It may beneficial to define it in this context.
  - Page 4 line 91-93
    *Family planning, according to the WHO, consists in practices that “allow people to attain their desired number of children, if any, and to determine the spacing of their pregnancies” [14].*

**Methods:**

- Research Study Design: In lines 115-117, there are no citations to support the description of prospective qualitative analysis. To avoid unintentional plagiarism, ensure all ideas that are not originally yours are cited.
  - Thank you for this sharp comment. We have added a reference to this paragraph.
    Page 5 line 132-135
    *A prospective qualitative research methodology was adopted to gain a deep understanding of women’s lived experiences over time [32]. A prospective study can best answer our research questions as participants can be interviewed during their pregnancy and after birth and therefore their experiences can be analyzed as they unfold [32].*
- In 118, a narrative approach was used, however, you fail to describe the appropriate of understanding the construction and meaning of the experience. This is different than phenomenological research from which you would extract meaning from shared experiences among these women to understand the essence of UP. Justification on the narrative approach for this population is warranted. It is unclear how this approach was used and how phenomenological underpinnings were incorporated in extracting meaning from these stories.
  - Thank you for your insightful comment to clarify our use of a narrative approach with phenomenological research. We meant that the interviews had a chronological narrative-style approach as they were performed during pregnancy and after birth. However, we understand the confusion we created by using this term, therefore we deleted it from the manuscript.
    Page 5 line 136
    *We conducted semi-structured interviews during and after pregnancy.*
    Page 9 line 222-223
    *Thus, it was possible to initially focus on the individual narratives before making interpretations at the group level.*
- Researcher Description: The researchers have thoroughly described their positions but did not disclose the intersection of other identities that may impact their knowledge of the phenomenon, responsiveness, and interpretation of the results. Positionality was alluded to but there is no reference to power dynamics that may have existed based on these identities, both with the study participants and amongst the researchers.
  - Thank you for your insightful recommendation. We agree with this and have now adapted the researcher description to address positionality from an intersectional lens.
    Page 6-7 line 147-169
    *The position of the researchers holds relevance in any study conducting IPA, as it adheres to the double hermeneutic position: the researchers attribute meaning to how the participants attribute meaning to their own experience [39]. NS is a medical doctor with experience in the field of obstetrics and perinatal psychiatry; HS is a midwife with special knowledge of and interest in international public health; ES is a medical doctor with experience in psychiatry and obstetric care for marginalized groups of pregnant women; ME is a postdoc researcher who has lived experience in perinatal psychiatry and is an employee of organization MIND (the Dutch mental health patient and family umbrella organization); NJ is a postdoc researcher with methodological expertise; MP is a senior researcher and perinatologist; TZ is a researcher and hospital psychiatrist; OH is a senior researcher, psychiatrist with expertise in hospital psychiatry and has lived experience with UPs and psychiatric vulnerability; and BB is a senior researcher and psychiatrist with perinatal expertise. As a research team, we also acknowledge that our identities and social positions may have influenced the research process. Most members of the team identify as women and have a clinical professional background which may have shaped our empathy toward pregnant participants as well as our assumptions about clinical care. Several researchers are parents themselves or have personal experience with psychiatric vulnerability or unintended pregnancy, which may have increased our sensitivity to participants’ accounts, but also required ongoing reflexivity to avoid over-identification. Despite diversity in cultural and socioeconomic backgrounds within the research team, all researchers occupy positions of relative educational and institutional privilege. This shared positionality may have contributed to power asymmetries in interactions with participants. Furthemore, two researchers (NS and ES) were junior researchers and PhD students, thus dependent on the more senior colleagues. We discussed these dynamics throughout data collection and analysis, and reflexively considered how our identities, professional roles, and lived experiences influenced the questions we asked, our interpretation of participants’ narratives, and the meaning-making process.*
- There is no description about reflexivity and methods used to address psychological and emotional responses to the data, bracketing, consultation, or other means for example to reduce bias. Failing to provide this information significantly impacts rigor and transparency. The researcher is also an instrument in qualitative inquiry.
  - Thanks for your useful comment. We did not make use of bracketing during the analysis, as we followed a methodology from Charlink et al [1]. We did make use of consultation and triangulation to reduce possible bias and increase credibility and transparency, and have now included this in the manuscript.

Page 8 line 223-225
*The last step included examining the analysis in a group meeting to reduce possible personal biases and enhance transparency (all authors). Credibility was therefore enhanced through investigator triangulation [43].*
Page 34 line 848-849
*Finally, while we made use of triangulation, we did not use bracketing to reduce authors’ biases.*

- Participant Selection and Recruitment: In line 139-140, the type of recruitment (e.g., snowball, convenience, purposive, etc.) needs to be specified.
  - Thanks for the sharp remark, we have elucidated the sampling later in this section.
    Page 7 line 186-188
    *Purposeful sampling enabled the inclusion of participants with UPs, a willingness to discuss the topic and varying psychiatric vulnerability. Moreover, snowball sampling was used to recruit the partners of the participants.*
- Review this segment as there are some missing punctuations and issues with grammar. For example, in line 147, the sentence should read, "Participants were asked..." instead of "was asked."
  - We apologize for these oversights and have made grammatical edits.

Page 7 line 180-181
*When pregnant women agreed to participate, patient information documents and informed consent forms were sent via email. Each pregnant woman was asked whether a partner was involved and if this partner wanted to be interviewed too, separately or together.*

- In line 147-149, clarity is needed on criteria for inviting persons to interviews and partners participating in interviews. Asking participants if their partners wished to participate is called snowball sampling which means you have used both purposive for your primary participants and snowball sampling for recruiting their partners. There is no description about partners receiving informed consent separate from their partner.
  - Thank you for your insightful feedback. We have adapted and clarified the sampling process.
    Page 7, line 179-188
    *When pregnant women agreed to participate, patient information documents and informed consent forms were sent via email. Each pregnant woman was asked whether a partner was involved and if this partner wanted to be interviewed too, separately or together. When the partner agreed to participate, they also signed an informed consent form. Participants and partners were invited for two consecutive interviews—one during pregnancy and one within three to six months postpartum. Participants received reimbursement for their time and travel expenses. Purposeful sampling enabled the inclusion of participants with UPs, a willingness to discuss the topic and varying psychiatric vulnerability. Snowball sampling was used to recruit the partners of the participants.*
- Data Collection: In line 159-160, describe your approach to maintaining confidentiality for persons interviewing at the hospital. For in-person interviews, what software or device was used to record the interviews?
  - Thank you for your observation.
    Page 7 line 193-196
    *Interviews were recorded by a voice recorder (not connected to internet, Bluetooth or Wifi). The voice recorder was started after an informal introduction of both interviewee, interviewer, and observer. After the interview had taken place, the recordings were uploaded to a secured hospital-based data management system.*
- Data Analysis: In line 164, specify if a third-party transcribed the interviews OR if the researchers transcribed the interviews independently. Did researchers review the transcriptions and reconcile discrepancies? This information is crucial in ensuring the participants experiences were captured.
  - Thank you. We elucidated the transcription process in more details.
    Page 8 line 208-210
    *The recorded interviews were manually anonymized and transcribed verbatim by the researchers (NS and ES) independently. The transcripts were checked a second time (NS and ES) for possible errors. We generated summaries and shared them with participants for credibility checks.*

**Results:**

- In line 187, there are noticeable differences in when follow-up interviews were conducted. In reviewing your methods section, the follow-up time for the second interview was not specified. State the intend follow-up time for the second interview (e.g., estimated 8-weeks postpartum) and if this was not accomplished, factors that influenced follow-ups.
  - Thank you for your remark. We invited every participant 6 weeks postpartum. The variation in the timing of the second interview was based on how quick the participant responded and their availability for an appointment. We elucidated this process in the methods and results.
    Page 7 line 184-186
    *Participants and partners were invited for two consecutive interviews—one during pregnancy and one within two to six months postpartum, depending on availability of the participant. The participant invitations were sent six weeks after birth.*
    Page 9 line 238-239
    *The variation in timing for the postpartum interview depended on the availability of the participant.*
- The remainder of the results were comprehensive, with select quotes accurately reflecting themes and sub-themes that emerged in the data. Quotes captured the essence of patients' experiences. The researchers did not attempt to interpret these findings and maintained a neutral position by articulating what participants' stated.
  - Thank you for your compliment.
- In lines in 659-681, the discussion section is not designed to restate what is in the results. This information should be interwoven into the interpretation section.
  - Thank you, we have condensed and integrated these sections. As it is quite a lengthy section despite the adaptations we have not added it here. You can find the adapted section on pages 29-32, line 728-796.
- Strengths & Limitations: In line 701-702, there appears to be a typo.
  - Thanks for finding the typo.
    Page 33 line 821-823
    *In our sample, all participants were employed, indicating a middle to high socioeconomic status, that may have influenced their ability to continue the pregnancy for financial reasons.*
- Clarify if you are referring to women of low- to middle-class or SES and how this can influence decisions in family planning.
  - Thank you for your comment, we further clarified this section.
    Page 33 line 821-829
    *In our sample, all participants were employed and living independently, indicating a middle to high socioeconomic status, that may have influenced their ability to continue the pregnancy for financial reasons. Beyond financial resources, higher socioeconomic status is associated with broader structural advantages, including better access to healthcare, stable housing, health literacy, and supportive social networks. As articulated in Ross’s framework of reproductive justice, access to healthcare and housing, as well as the presence of a support system, are fundamental factors—and basic human rights—when considering whether to continue an unintended pregnancy [55]. In samples with fewer socioeconomic resources, financial insecurity and related structural barriers may therefore play a more prominent role in shaping pregnancy-related decision-making.*
- There is no mention of instrument limitations for other methodological limitations. For example, how did modifying the semi-structured interview possibly impact responsiveness to prompts and data that was obtained? Examine the limitations of narrative and phenomenological inquiry and the sensitivity of this topic.
  - We thank the reviewer for this comment. We have revised the manuscript to explicitly address instrument-related and methodological limitations. The semi-structured interview guide was iteratively adapted during data collection, which may have influenced responsiveness to specific prompts and introduced variability in the depth and emphasis of topics across interviews. This flexibility supported participant-led narratives. In addition, we acknowledge limitations inherent to narrative and phenomenological inquiry, including reliance on retrospective meaning making and participants’ ability to verbalize complex, embodied experiences. Given the sensitive nature of the topic, self-censorship, social desirability, and power dynamics may also have shaped what participants felt able to disclose. These considerations are now explicitly discussed in the Methods and Limitations sections as follows.
    - Page 33 line 837-849

*Because some interviews were conducted with partners present while others were conducted individually, the presence or absence of support persons may have influenced participants’ willingness to disclose sensitive emotional experiences or relationship dynamics, potentially shaping the depth and direction of the narratives. In addition, the interview guide was iteratively adapted throughout data collection. While this flexibility supported participant-led storytelling and allowed emerging issues to be explored, it may also have affected participants’ responsiveness to specific prompts and introduced variability in the salience and depth of topics across interviews, thereby influencing the nature of the data obtained. Furthermore, there is a possibility of response bias (socially acceptable responses), but we consider this bias limited because the participants’ responses were diverse and captured a range of emotions and reactions. Several issues are related to the data collection. Diversity in the timing of the interview (weeks of gestation) might have increased recall bias. Finally, while we made use of triangulation, we did not use bracketing to reduce authors’ biases.*

- Furthermore, there is no description of how the presence or absence of partners and support persons may have influenced responses during the interviews.
  - Thank you for your comment. We have added this in the limitations.
    Page 33 line 837-840
    *Because some interviews were conducted with partners present while others were conducted individually, the presence or absence of support persons may have influenced participants’ willingness to disclose sensitive emotional experiences or relationship dynamics, potentially shaping the depth and direction of the narratives.*

**General:**

- Ensure the interview guide is available in an appendix.
  - Thank you, we have added the interview guide in the appendix, (see page 47-48).

**Author’s comments**

- Page 11 line 248
  A few mistakes were noticed in the table, therefore a new version has been added.
